# A Rare Case of Bilateral Xanthogranulomatous Pyelonephritis Secondary to Candida albicans

**DOI:** 10.7759/cureus.78131

**Published:** 2025-01-28

**Authors:** Prakrati Yadav, Ji Yoon Park, Aijaz Parisa

**Affiliations:** 1 Internal Medicine, Charleston Area Medical Center, Charleston, USA

**Keywords:** candida albicans, fungal infection, nephrectomy, renal enlargement, xanthogranulomatous pyelonephritis

## Abstract

Xanthogranulomatous pyelonephritis (XGP) is a rare, chronic inflammatory renal disease typically caused by bacterial infections. We hereby report a rare case of bilateral XGP secondary to Candida albicans in a 31-year-old female with multiple autoimmune disorders. The patient presented with nonspecific abdominal discomfort, painful urination, and generalized weakness. Computed tomography (CT) of the abdomen revealed bilateral hydronephrosis, renal enlargement, and chronic changes consistent with XGP. Despite aggressive antifungal therapy, her renal function progressively deteriorated, ultimately leading to multi-organ failure and death. This case underscores the importance of maintaining a high index of suspicion for XGP in immunocompromised patients to facilitate early diagnosis and potentially improve outcomes. Additionally, it highlights the unique diagnostic challenge posed by C. albicans as a causative pathogen in XGP, emphasizing the need to consider fungal infections in the differential diagnosis to avoid delays in treatment.

## Introduction

Xanthogranulomatous pyelonephritis (XGP) is a rare form of chronic pyelonephritis characterized by granulomatous inflammation of the renal parenchyma. It most often results from long-standing urinary tract obstruction and recurrent bacterial infections, typically due to Escherichia coli and Proteus mirabilis [1]. XGP often poses a diagnostic challenge due to nonspecific signs and symptoms and often mimics other differentials on imaging, including renal carcinoma, abscess, tuberculosis, lymphoma, angiolipoma, leiomyosarcoma, megalocytic interstitial nephritis, malakoplakia, or Wilms tumor [2]. Therefore, early recognition is crucial, as the definitive treatment is nephrectomy. If untreated, XGP can lead to severe complications, including sepsis and renal failure, with a mortality rate of approximately 10% [3]. XGP is usually unilateral and bilateral involvement is exceedingly rare, with only a handful of cases reported in the literature [4,5]. Furthermore, fungal infections such as Candida albicans have rarely been associated with XGP [6]. We report a rare case of bilateral XGP secondary to C. albicans in a patient with multiple autoimmune conditions.

## Case presentation

A 31-year-old woman, with a complex medical history, including systemic lupus erythematosus (on mycophenolate mofetil and hydroxychloroquine), type 1 diabetes mellitus, autoimmune pancreatitis, chronic pancreatitis, esophageal stricture, and malnutrition, presented to the emergency department with complaints of generalized weakness, decreased oral intake, dysuria, and left flank abdominal pain that had worsened over the previous month. On examination, the patient appeared ill with a BMI of 11.6 kg/m^2^ but not acutely distressed. Laboratory results (Table 1) revealed elevated white blood cell count, creatinine, and C-reactive protein levels.

**Table 1:** Laboratory values on presentation

	Patient	Reference values
White Blood Cell	22.3 × 10⁹/L	4.8-10.8 × 10⁹/L
Hemoglobin	13.5 g/dL	12-16 g/dL
Platelet	325,000/dL	140,000-450,000/dL
Segmented Neutrophils	74%	50-70%
Band Neutrophils	24%	0-8%
Lymphocytes	1%	20-35%
Eosinophils	1%	0-4%
Basophils	0%	0-2%
Monocytes	1%	1-13%
Blood Urea Nitrogen	101 mg/dL	7-25
Creatinine	3.1 mg/dL	0.6-1.2 mg/dL
Alanine Aminotransferase	35 U/L	7-52 U/L
Aspartate Aminotransferase	22 U/L	13-39 U/L
Alkaline Phosphatase	163 U/L	34-104 U/L
Total Protein	7.9 g/dL	6.4-8.2 g/dL
Albumin	3.2 g/dL	3.4-5.0 g/dL
Total Bilirubin	0.3 mg/dL	0.3-1.0 mg/dL
Direct Bilirubin	0.28 mg/dL	0.03-0.18 mg/dL
C-Reactive Protein	173.6 mg/L	<5.0 mg/L
Serum Glucose	214 mg/dL	74-106 mg/dL
Hemoglobin A1C	11.9%	<5.7%
HIV	Negative	-
Hepatitis B Surface Antigen	Negative	-
Hepatitis C Virus Antibody	Negative	-
Serum HCG, Quantitative	Negative	-

**Figure 1:**
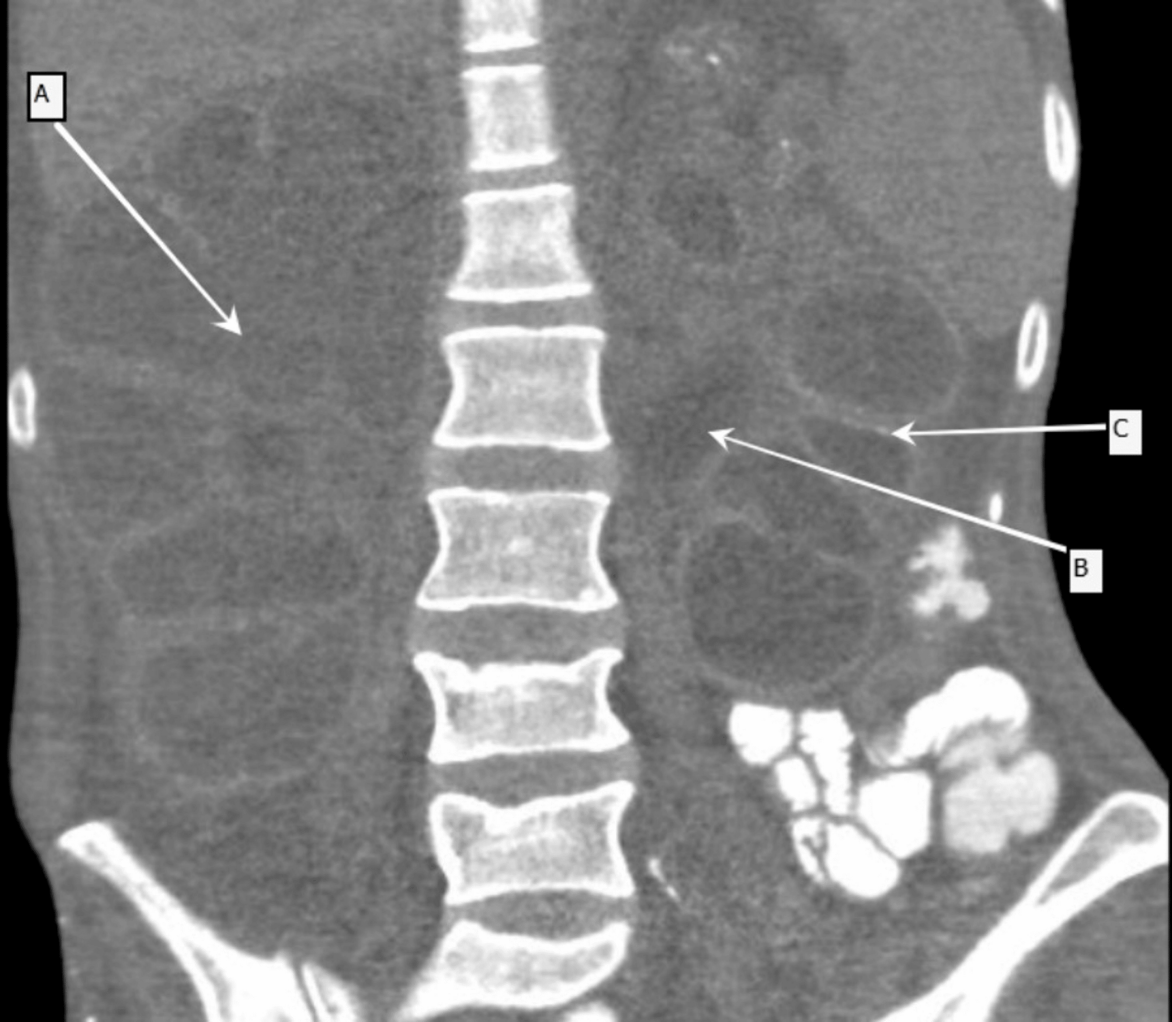
Non-contrast computed tomography of the abdomen Coronal section showing markedly enlarged bilateral renal parenchyma with dilated calyces [A], left-sided severe hydronephrosis [B], and calcifications in the junctions of the corticomedullary junctions of the kidneys bilaterally [C].

Urinalysis showed significant leukocyte esterase. Blood and urine cultures on admission were negative. A review of previous imaging indicated bilateral hydronephrosis and renal enlargement dating back to four months prior to the current admission. During her current admission, contrast-enhanced abdominal CT (Figure 1) revealed diffuse nephromegaly, extensive corticomedullary calcifications, chronic cystic changes, and bilateral hydronephrosis, which raised concern for XGP.

A left-sided ureteral stent was placed by urology to alleviate obstruction, and urine cultures obtained at the time of stent placement detected C. albicans using the Matrix-assisted laser desorption ionization-time of flight (MALDI-TOF) mass spectrometry assay with antifungal susceptibilities, as shown in Table 2.

**Table 2:** Antifungal susceptibility of Candida albicans detected in urine culture

	Candida albicans	
Drug	MIC Interpretation	MIC Dilution
Amphotericin B	-	0.5
Anidulafungin	S	0.06
Caspofungin	S	0.128
Fluconazole	S	0.25
Voriconazole	S	0.008

The patient was started on fluconazole 200 mg daily. Despite antifungal therapy, her renal function continued to deteriorate, with creatinine rising to 5.3 mg/dL (normal range: 0.6-1.2 mg/dL) and minimal urine output. 

Bilateral nephrostomy tubes were inserted for further drainage and source control, with a yellowish, pus-like output. Repeated cultures from the nephrostomy tubes grew C. albicans, and her condition continued to worsen despite two weeks of fluconazole. Eraxis (anidulafungin) was added for broader antifungal coverage. Intravenous conventional amphotericin B (AmB) and irrigation through nephrostomy tube with AmB were considered but could not be implemented due to non-availability. Eraxis (anidulafungin) was added along with fluconazole to broaden antifungal coverage due to limited available options. Given her complex medical history and high surgical risk (National Surgical Quality Improvement Program (NSQIP) risk of serious complications 35%), nephrectomy was deemed too risky after discussion with the patient and her family.

The patient developed severe complications, including fluid overload with bilateral pleural effusions, chronic malnutrition, aspiration pneumonia, and sepsis. After extensive discussions, the patient opted for comfort care, and she subsequently passed away.

## Discussion

XGP is a rare and often misdiagnosed form of pyelonephritis that typically results from chronic bacterial infections in the context of obstructive uropathy [1]. The pathogenesis of XGP involves a combination of immune response, chronic infection, and inflammation leading to the destruction of renal parenchyma. While P. mirabilis and E. coli are the most common pathogens in bacterial cases, fungal infections, particularly those caused by C. albicans, have been rarely reported as a cause of XGP [6,7].

Risk factors for XGP include ureteropelvic junction syndrome, ureteropelvic duplication, vesicoureteral reflux, and bladder cancer. Systemic risk factors include diabetes, metabolic syndrome, immunosuppression, rheumatoid arthritis, and other chronic inflammatory states [2]. In the case presented, the patient’s comorbidities, including diabetes and autoimmune disorders with ongoing malnutrition, compounded her susceptibility to XGP. This underscores the importance of recognizing systemic risk factors in predisposing individuals to uncommon renal pathologies. C. albicans, an opportunistic pathogen, is not typically associated with XGP, but it can infect the renal parenchyma in immunocompromised individuals [8]. To our knowledge, bilateral XGP secondary to C. albicans has not been previously described in the literature, making this case particularly unique.

XGP involves granulomatous inflammation leading to the destruction of the affected kidney and the formation of grossly distended yellow, pus-filled nodules in the renal calyces. Histologically, it involves the replacement of renal parenchyma by foamy, lipid-laden macrophages called xanthoma cells [2]. XGP is usually unilateral and bilateral involvement is rare, with only a few cases documented in the literature. A systematic review by Harley et al. found only four reported cases of bilateral XGP out of more than 1,000 total cases [6].

Computed tomography (CT) is the mainstay of diagnostic imaging as appearances on ultrasound and MRI can be non-specific. Imaging findings in XGP include renal enlargement, perinephric inflammation, collections or abscesses, dilated calyces, and abnormal parenchyma [2,9]. XGP can be classified into three stages based on imaging findings: Stage 1 is confined to the renal parenchyma, Stage 2 is the involvement of renal parenchyma and peri-nephric fat, and Stage 3 is involvement para-renal space/retroperitoneum [10]. Although a definite diagnosis is made by histology, clinical presentation and radiological findings can point toward the diagnosis of XGP in most cases [2]. Although the histological diagnosis was not established in this case, the yellow thick pus discharge from nephrostomy tubes, which is seen on the gross specimen of the resected kidney with XGP [11] and characteristic imaging findings supported the diagnosis of XGP.

The management of XGP typically involves nephrectomy and antimicrobial therapy [12]. Although most cases are bacterial, the treatment of fungal-associated XGP presents challenges due to the difficulty in achieving source control while managing the fungal infection. In this case, despite initial antifungal therapy with fluconazole, the patient’s renal function continued to decline, highlighting the difficulties in treating such infections in immunocompromised individuals. Amphotericin B (AmB) deoxycholate is usually recommended in pyelonephritis secondary to fluconazole-resistant species of candida with or without flucytosine [13]. For patients with nephrostomy tubes, irrigation with AmB deoxycholate is also recommended [13]. Although echinocandins have minimal excretion of active drugs into the urine and are usually not recommended for candida urinary tract infections; it has been seen to achieve good tissue concentrations in infections localized to the kidneys [13]. It was used in conjunction with fluconazole in our case to provide dual anti-fungal coverage due to the non-availability of AmB deoxycholate.

This case also underscores the importance of early suspicion of XGP based on imaging and early nephrectomy for definitive treatment to prevent potentially fatal complications as untreated cases can result in a mortality rate of approximately 10% [3]. It also highlights the need for a multidisciplinary approach, involving urologists, infectious disease specialists, and interventional radiologists, to manage these complex cases.

## Conclusions

Bilateral XGP secondary to C. albicans is a rare and challenging condition, particularly in patients with multiple comorbidities. Early recognition based on imaging and microbiological data is crucial for effective management and to prevent complications. The case emphasizes the need for individualized treatment plans, especially in immunocompromised patients, and the importance of considering fungal infections in the differential diagnosis of XGP.
